# Turn It Down a Notch

**DOI:** 10.3389/fcell.2016.00151

**Published:** 2017-01-18

**Authors:** Francesca A. Carrieri, Jacqueline Kim Dale

**Affiliations:** Division of Cell and Developmental Biology, School of Life Sciences, University of DundeeDundee, UK

**Keywords:** somitogenesis, embryonic development, signalling pathway, notch, FBXW7

## Abstract

In the developing vertebrate embryo, segmentation initiates through the formation of repeated segments, or somites, on either side of the posterior neural tube along the anterior to posterior axis. The periodicity of somitogenesis is regulated by a molecular oscillator, the segmentation clock, driving cyclic gene expression in the unsegmented paraxial mesoderm, from which somites derive. Three signaling pathways underlie the molecular mechanism of the oscillator: Wnt, FGF, and Notch. In particular, Notch has been demonstrated to be an essential piece in the intricate somitogenesis regulation puzzle. Notch is required to synchronize oscillations between neighboring cells, and is moreover necessary for somite formation and clock gene oscillations. Following ligand activation, the Notch receptor is cleaved to liberate the active intracellular domain (NICD) and during somitogenesis NICD itself is produced and degraded in a cyclical manner, requiring tightly regulated, and coordinated turnover. It was recently shown that the pace of the segmentation clock is exquisitely sensitive to levels/stability of NICD. In this review, we focus on what is known about the mechanisms regulating NICD turnover, crucial to the activity of the pathway in all developmental contexts. To date, the regulation of NICD stability has been attributed to phosphorylation of the PEST domain which serves to recruit the SCF/Sel10/FBXW7 E3 ubiquitin ligase complex involved in NICD turnover. We will describe the pathophysiological relevance of NICD-FBXW7 interaction, whose defects have been linked to leukemia and a variety of solid cancers.

## Introduction

The formation of a segmented body plan is a conserved feature of embryogenesis for all vertebrate species. This process leads to the formation of transient embryonic segments, called somites. Somites are precursors of vertebrae and ribs, associated skeletal muscles, and some dermis (Christ et al., 2007). Their formation is regulated by a molecular oscillator called the segmentation clock (Gibb et al., 2010; Oates et al., 2012; Benazeraf and Pourquie, 2013). Aberrations in this mechanism lead to human developmental disorders, such as spondylocostal dysostosis (Pourquie, 2011; Eckalbar et al., 2012). Some of these malformations originate from defects in Notch signaling, suggesting that this pathway is essential in controlling and regulating vertebrate segmentation.

This review aims to give a general overview of the importance of the Notch signaling pathway in the segmentation clock in addition to a description of our current understanding of the Notch pathway, particularly focusing on the turnover and regulation of the Notch intracellular domain.

## Somitogenesis

Somitogenesis has been the topic of several outstanding reviews (Pourquie, 2001; Maroto et al., 2012; Oates et al., 2012; Benazeraf and Pourquie, 2013; Hubaud and Pourquie, 2014; Bailey and Dale, 2015), thus we will provide a general overview.

**Figure 1:**
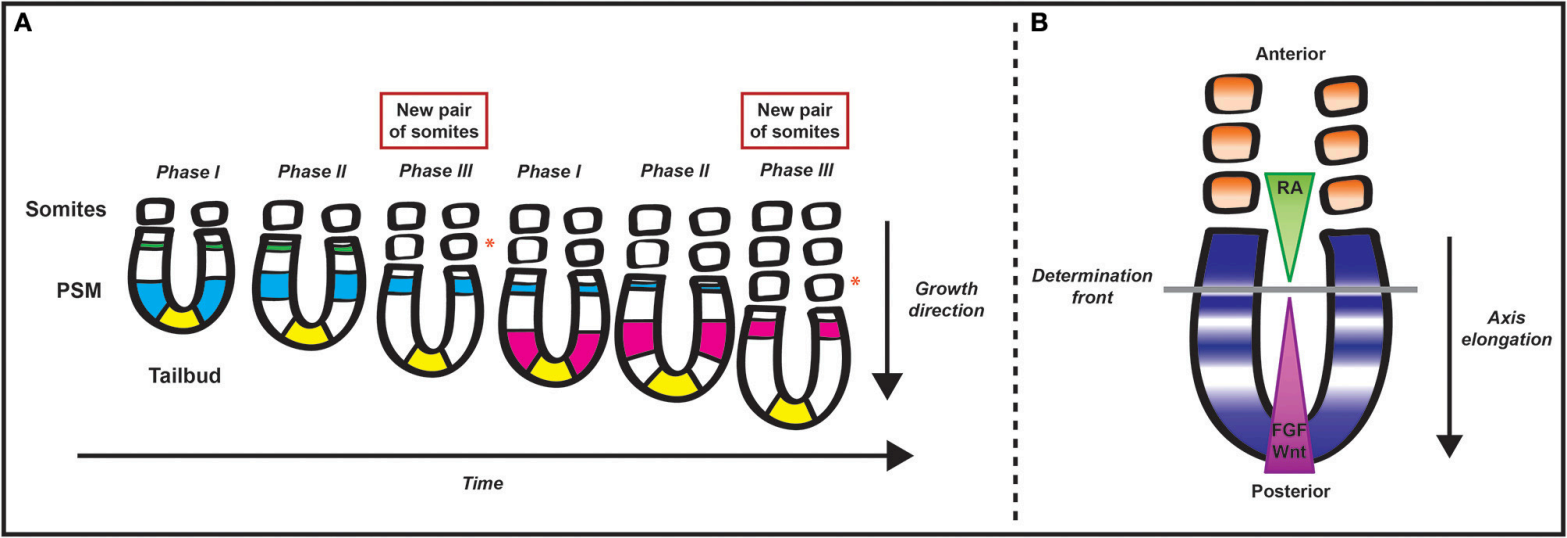
**Schematic representation of somitogenesis and the segmentation clock**. **(A)** Pairs of somites bud off from the rostral end of the presomitic mesoderm (PSM) progressively during early development. The tail bud, a site of gastrulation that lies at the posterior end of the embryo, continuously “replenishes” the posterior end of the PSM with progenitor cells. The periodicity of segmentation is regulated by a molecular oscillator that drives cyclic gene expression from the posterior to the anterior tip of the PSM. The different colors represent domains of clock gene expression in different cycles. As time progresses in each cycle, the domain of clock gene expression shifts anteriorly while narrowing until it reaches the anterior limit of the PSM. The periodicity of this cyclic gene expression matches that of somite formation. An orange asterisk lies adjacent to each of the new pairs of somites formed in the time series—the first pair is formed after the blue wave of clock gene expression traverses the PSM and the second pair is formed after the pink wave of clock genes expression traverses the PSM from the tail bud to the anterior limit of the tissue. **(B)** Two mutually opposing gradients of retinoic acid (RA) and FGF/Wnt regulate the maturation wavefront within the paraxial mesoderm. Due to somite formation anteriorly and gastrulation at the caudal end of the PSM, cells within the PSM become progressively more anteriorly displaced, and, as a result, they are exposed to progressively lower levels of FGF/Wnt. There is a position within the PSM, termed the determination front, where cells are released from the effect of FGF and can respond to the segmentation clock and RA, embarking on their segmentation programme.

Early in development, segmentation initiates through the formation of repeated segments, or somites (Christ et al., 2007; Gibb et al., 2010). Somitogenesis is a cyclical and gradual process such that somites are sequentially pinched off in pairs from the anterior end of two rods of paraxial mesoderm, the presomitic mesoderm (PSM), lying on either side of the caudal neural tube (Gossler and De Angelis, 1998; Cambray and Wilson, 2007; Dequeant and Pourquie, 2008; Gibb et al., 2010; Maroto et al., 2012). The PSM is continuously replenished with progenitor cells located initially in both the epiblast adjacent to the primitive streak and the rostral primitive streak and later in the tail bud (Iimura et al., 2007; Gomez and Pourquie, 2009; Henrique et al., 2015), and thus the presomitic mesoderm preserves its length (Dequeant and Pourquie, 2008; Figure 1A).

The periodicity of this segmentation process is different from species to species: 30 min in zebrafish (Schroter et al., 2008), 90 min in chicken (Palmeirim et al., 1997), 2 h in mice (Tam, 1981), 6–8 h in human (William et al., 2007). Similarly, the total number of somites is a characteristic feature of each species: 31 pairs in zebrafish, 50 somite pairs in chicken, 65 in mice, and about 500 in some snakes.

The regulation of the periodicity of somitogenesis is governed by the segmentation clock, a molecular oscillator (Palmeirim et al., 1997) whose existence was first proposed in theoretical models such as the “*Clock and Wavefront model”* (Cooke and Zeeman, 1976). According to the model, a wavefront of maturation sweeps along the body axis concomitant with extension of the trunk and tail, governing maturation of the PSM to become somites. This positional information gradient within the PSM interacts with a smooth cellular oscillator (the clock), driving cells to oscillate between a permissive and a non-permissive state. Segmentation of the PSM only occurs when the maturation wavefront reaches a group of cells in a specific “permissive” clock phase (Cooke and Zeeman, 1976).

Over the last 20 years the theoretical “*Clock and Wavefront model”* has received significant experimental support. The wavefront of maturation is thought to rely on the intersecting gradients and cross-regulatory activities of three signal pathways, namely a caudo-rostral gradient of FGF and Wnt and rostro-caudal gradient of retinoic acid (RA). The determination front marks the point of intersection of these gradients, where the next prospective somite boundary will form (Figure 1B). These cross-regulatory activities thereby regulate somite size. The activity of Wnt and FGF also controls cell maturation in the PSM. These roles have been reviewed elsewhere, thus will not be covered here (Aulehla et al., 2003; Dubrulle and Pourquie, 2004; Wahl et al., 2007; Aulehla and Pourquie, 2010; Hubaud and Pourquie, 2014).

It is well established that the rhythmicity of somitogenesis is regulated by the segmentation clock driving cyclic and dynamic expression of “clock genes” in the PSM, with a periodicity that matches somite formation. This feature is conserved among a variety of vertebrate species (Jiang et al., 2000; Cinquin, 2007; Dequeant and Pourquie, 2008; Gomez et al., 2008; Ozbudak and Lewis, 2008; Krol et al., 2011). The clock genes are components of the Notch, Wnt, and FGF pathways (Aulehla et al., 2003; Dequeant and Pourquie, 2008; Yabe and Takada, 2016), playing a reciprocal regulatory role in oscillatory gene expression (reviewed in Gibb et al., 2010; Maroto et al., 2012). While the specific genes which oscillate may vary among species, the most highly represented pathway among the clock genes is the Notch (Krol et al., 2011).

Stemming from the observation that the proteins encoded by clock genes are predominantly unstable negative regulators of the pathway that activates them, it is believed that oscillatory gene expression relies on negative feedback loops of these unstable regulators, such as the two Notch target clock genes, *Hes7*, and *Lunatic Fringe* (Lfng) (Bessho et al., 2001a,b, 2003; Cole et al., 2002; Hirata et al., 2002; Dale et al., 2003; Serth et al., 2003; Kageyama et al., 2012; Okubo et al., 2012). It is particularly interesting that blocking *Lfng* oscillations disturbs somitogenesis in the thoracic and lumbar areas but not in more posterior areas of the embryo (Shifley et al., 2008), implying the role of Notch signaling in segmentation is not uniform along the axis.

In addition to negative feedback, oscillatory gene expression in the PSM also invokes positive feedback; Notch signaling regulates dynamic expression of *Notch1* itself, whereas Wnt regulates dynamic expression of *Dll1* (Bone et al., 2014).

As the most highly conserved pathway involved in the segmentation clock, a wealth of studies have focused on elucidating the fundamental role of Notch in somitogenesis and in the segmentation clock mechanism (Barrantes et al., 1999; Jiang et al., 2000; Bessho et al., 2001b, 2003; Dale et al., 2003; Julich et al., 2005; McGrew et al., 2008; Hubaud and Pourquie, 2014; Wahi et al., 2014; Liao and Oates, 2016). Notch is clearly required to synchronize oscillations between neighboring cells (Jiang et al., 2000; Shimojo et al., 2016). A question that arises is whether oscillations are actually necessary for the segmentation process to occur or whether just non-oscillatory activity of the Notch pathway is sufficient. Mutant mice or fish lacking Notch components all display severe segmentation defects (Conlon et al., 1995; Barrantes et al., 1999; Jiang et al., 2000; Liao and Oates, 2016). For example, the lack of the obligate transcription factor *RBP-J*κ, in mouse, leads to lethality before day E10.5 and only the first few cervical somites are formed (Oka et al., 1995). A pivotal study conducted by Ferjentsik et al. pointed out that Notch activity, *per se*, is indeed essential for somite formation. Mutating crucial Notch pathway components, or using a complementary pharmacological approach, they demonstrated that in mouse Notch activity is crucial for the oscillatory activity of all clock genes, and thus essential for the formation of a segmented body axis (Ferjentsik et al., 2009) (see also Huppert et al., 2005).

## Notch signaling pathway

The Notch pathway is highly conserved among metazoans and mediates short range juxtacrine communication. The Notch locus was first cloned in *Drosophila* and it was found to encode a large single pass type I transmembrane protein (Wharton et al., 1985), whose epidermal growth factor (EGF) repeats mediate interaction with their canonical activators—two ligands, Delta, and Serrate, in the Delta-Serrate-Lag2 (DSL) family. *Drosophila* studies have contributed hugely to our current understanding of Notch (Artavanis-Tsakonas et al., 1999). The role of Notch in developmental processes of multicellular species has been extensively elucidated (Dumortier et al., 2005; Radtke et al., 2005; Aster, 2014). Notch signaling outcome mostly relies on the cellular context, and thus Notch affects stem cell maintenance, cell fate choice, cell differentiation, lineage progression, and apoptosis in a context-dependent fashion (Bray, 2006; Hori et al., 2013).

Despite its multiple roles and versatility, the Notch pathway is relatively simple and conserved across species (Artavanis-Tsakonas et al., 1999; Bray, 2006; Kopan and Ilagan, 2009). In mammals, there are four Notch receptors (NOTCH1-4) and five DSL ligands (JAG1-2 and Delta-like 1-3-4). Both receptors and ligands are single transmembrane proteins and thus to trigger the signaling cascade, cell-cell contact is required (D'souza et al., 2010; Andersson et al., 2011; Greenwald and Kovall, 2013).

**Figure 2:**
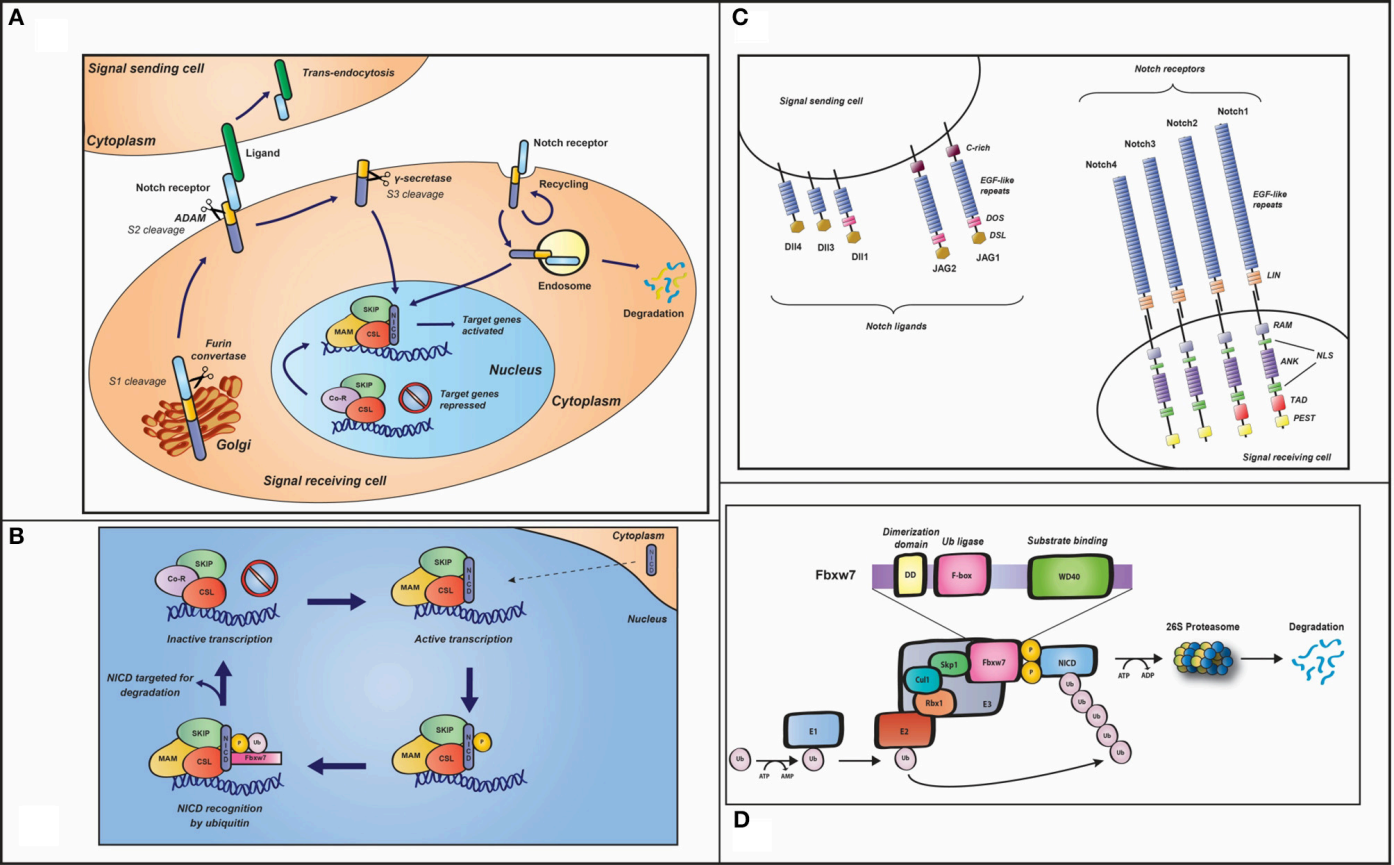
**(A)** The Notch signaling pathway. In the Golgi, after being glycosylated by members of the Fringe family, pre-Notch is cleaved by a Furin-like convertase into the extracellular and intracellular domains (termed the S1 cleavage), resulting in a heterodimeric receptor with non-covalently associated domains that is transported to the plasma membrane. The Fringe modifications introduced in the Golgi affect sensitivity of the receptor on the signal-receiving cell to the DSL (Delta-Serrate-Lag2) ligands, in the signal-sending cell. Following ligand-receptor interaction, trans-endocytosis of the Notch extracellular domain, by the signal-sending cell, exposes the second so called S2 cleavage site, facilitating intramembranous cleavage by an ADAM (a disintegrin and metalloproteinase domain) protease, in the extracellular domain. S2 cleavage, in turns, exposes the S3 proteolytic cleavage site within the transmembrane domain, which is cleaved by the γ-secretase complex and liberates the intracellular domain of Notch (NICD), allowing it to translocate to the nucleus and thus activate transcription of target genes. In order to prevent inappropriate signaling from the pool of Notch that has not been activated by ligand, Notch receptor is continuously internalized into early endosomes and thus degraded. **(B)** Zoom-in into the nucleus of the signal-receiving cell **(A)**. Once released into the nucleus, NICD binds the DNA-binding protein CSL as well as the SKIP protein. The trimeric complex thus recruits Mastermind-like protein (MAM), which recruits additional co-activators (not shown), required for the transcriptional regulation of Notch target gene expression. Kinases, such as CDK8 and GSK3β, phosphorylate (p) NICD on its PEST domain, rendering it susceptible to recognition by Fbxw7 E3 ligase, leading to ubiquitination (Ub) and subsequent degradation by the proteasome. In the absence of NICD, CSL associates with transcriptional co-repressors blocking Notch target gene activation. Target genes are repressed until more NICD is produced to re-initiate a new cycle of target gene expression. **(C)** Notch ligands and receptors. In the signal-receiving cell, the four mammalian Notch receptors (Notch1-4) are represented. They are expressed on the cell surface as heterodimers and characterized by epidermal growth factor (EGF)-like and LIN repeats in their extracellular region. The intracellular domain includes an RBP-Jκ-associated molecule (RAM) domain, seven ankyrin (ANK) repeats, two nuclear localization signals (NLS), a transactivation (TAD) domain (lacking in Notch3 and Notch4), and a Proline-, Glutamate-, Serine-, and Threonine-rich (PEST) domain. The five Notch ligands (Delta-like 1, 3, and 4 and Jagged 1 and 2) are represented on the surface of the signal-sending cell. Each ligand contains an EGF-like repeat region and a conserved domain DSL (Delta/Serrate/Lag). A conserved cysteine-rich (CR) domain is also present on Jagged1 and Jagged2. The DOS (Delta and OSM-11) domain, containing two atypical EGF repeats, is part of Dll1, JAG1 and JAG2 ligands. **(D)** SCF^Fbxw7^ E3 ubiquitin ligase complex representation. The upper part of the figure shows Fbxw7 domains: a conserved dimerization motif, which mediates dimerization of the SCF complex and thus facilitates ubiquitin conjugation; the F-box, which binds the SCF complex through Skp1; the WD40, containing three specific amino acid residues, which binds the phosphorylated substrate. In the bottom part of the figure the SCF^Fbxw7^ complex is schematically represented. In general, in the ubiquitin system, three enzymes are involved in the signaling cascade: the ubiquitin-activating E1, the ubiquitin-conjugating E2 and an E3 ubiquitin ligase. The first step is ATP-dependent and involves the binding of ubiquitin to E1. Ubiquitin is then activated and transferred to E2. The ubiquitin-E2 complex then interacts with a specific E3 (SCF^Fbxw7^, refer to main text for a description), which recognizes the substrate (phosphorylated NICD, in this case) and facilitates transfer of the ubiquitin molecules to the substrate, leading to substrate degradation by the 26S proteasome.

The Notch receptor is typically comprised of: (i) 29–36 EFG-like repeats in its extracellular domain, involved in ligand interaction; (ii) three juxtamembrane repeats (Lin-12-Notch, LIN), required for extra-intracellular domain interaction (located within the Negative Regulatory Region (NRR); (iii) the intracellular region, including seven ankyrin (ANK) repeats flanked by a PEST [rich in proline (P), glutamic acid (E), serine (S) and threonine (T) residues] and a transactivation (TAD) domain (Figure 2C).

During its maturation, Notch undergoes ligand-independent cleavage by a furin-like convertase in the *trans*-Golgi (Artavanis-Tsakonas et al., 1999; Fiuza and Arias, 2007; Hori et al., 2013). This first cleavage (the S1) results in the production of a heterodimeric receptor comprised of a transmembrane/intracellular fragment non-covalently bound to the Notch extracellular domain (NECD). Notch is thus presented to the cell surface as a heterodimer. The non-activated Notch receptor is constitutively internalized, ubiquitinated by Itch/AIP4 (a member of the Nedd4 family of HECT domain E3 ubiquitin ligases), and thus targeted for lysosomal degradation (Chastagner et al., 2008; Moretti and Brou, 2013).

To ensure correct folding and activity, during synthesis and secretion in the Golgi, NECD undergoes O-linked glycosylation and fucosylation (Rana and Haltiwanger, 2011). These two modifications on the EGF repeats modulate Notch activity by modulating interaction with the Delta or Serrate ligands. The reaction is catalyzed by three Fringe homologs (Lunatic, Manic, and Radical Fringe), recognizing specific amino acids in individual EGF repeats (Rampal et al., 2005). *In vitro*, in the signal-receiving cell, all Fringes enhance Dll1-Notch1 interactions with comparable effects in both *trans*- and *cis*-(Lebon et al., 2014). Rfng also enhances *trans*- and *cis*-interactions between JAG1 and Notch1, but these interactions are weakened by Lfng and Mfng. By contrast, JAG1 activation of Notch2 is potentiated by Lfng, thereby expanding the ligand-receptor combinations that are differentially modified by the Fringe enzymes (Hicks et al., 2000). In the context of somitogenesis, Lfng is the only family member expressed in the PSM. In most systems, Lfng acts in the receiving-cell to potentiate receptor activation by Delta-like ligands while reducing activation by Jagged ligands (Hicks et al., 2000; Yang et al., 2005; Kato et al., 2010). However, it has been suggested that LFNG protein may synchronize clock oscillations between neighboring cells by acting in the signal-sending cell to inhibit Notch1 activation by Dll1 (Okubo et al., 2012). Ligand binding in an adjacent cell triggers a second cleavage, mediated by the metalloprotease ADAM10 (A disintegrin and metalloprotease) at S2 site in the juxtamembrane extracellular domain, proximal to the Notch transmembrane domain (Mumm et al., 2000; Dyczynska et al., 2007; Bozkulak and Weinmaster, 2009; Gordon et al., 2009; Van Tetering et al., 2009; Weber et al., 2011; Groot et al., 2014). The cleaved NECD product, bound to the ligand, undergoes trans-endocytosis into the ligand-expressing cell (Kramer, 2000; Parks et al., 2000; Meloty-Kapella et al., 2012). The second cleavage exposes the third cleavage site, S3, within the membrane-tethered Notch fragment, and is thus a rate-limiting step for the third and final cleavage (Brou et al., 2000; Mumm et al., 2000). Upon cleavage at the S3 site by a γ-secretase complex, the Notch intracellular domain (NICD) is then released (Schroeter et al., 1998) and translocates into the nucleus to activate transcription of target genes (Figure 2A). Notch can be activated in the endosomal pathway, independently of its ligands, through the activity of Deltex, a Ring-domain ubiquitin ligase that binds to NICD. However, it is unclear how the Deltex-activation mechanism relates to that of ligand-induced signaling.

Notch signaling does not require the use of second messengers. The activity is exclusively driven by nuclear concentration of NICD (Struhl and Adachi, 1998; Ehebauer et al., 2006). In the nucleus, NICD binds a bi-functional transcription factor CSL [CBF1, Su(H), Lag-1], a DNA binding complex Mastermind (MAM), and a variety of other co-activators involved in the transcriptional activation of Notch target gene expression (Fryer et al., 2004; Kopan and Ilagan, 2009; Hori et al., 2013). The transcriptional co-regulator SKIP (Ski-interaction protein) and the histone acetylase p300 are recruited concomitantly to the promoter region of target genes promoting the assembly of the initiation and elongation complexes (Zhou et al., 2000; Wallberg et al., 2002; Fryer et al., 2004; Bray, 2006; Figure 2B). MAM also engages kinases that phosphorylate NICD (Wu et al., 2000; Kitagawa et al., 2001; Nam et al., 2003; Fryer et al., 2004), a crucial step in the regulation of NICD stability and activity (Ingles-Esteve et al., 2001; Espinosa et al., 2003; Fryer et al., 2004; Jin et al., 2009). The domain targeted is the C-terminal PEST domain that is phosphorylated by the cyclin C cyclin-dependent kinase-8 complex (Cyc:CDK8) and glycogen synthase kinase 3β (GSK-3β) (Espinosa et al., 2003; Fryer et al., 2004; Jin et al., 2009).

## FBXW7 and its role in NICD turnover

NICD phosphorylation leads to its ubiquitination, turnover, and degradation by the proteasome, defining the half-life of Notch signaling, allowing the cell once again to become ligand-competent and resetting the signaling for a new cycle of activation (Le Bras et al., 2011). In the prevailing model, the ubiquitin ligase involved is the SCF^Fbxw7^ [S phase kinase-associated protein 1 (SKP1)-Cullin 1 (CUL1)-F-box] protein complex (Wu et al., 2001; Tsunematsu et al., 2004; Crusio et al., 2010). SCF^Fbxw7^ is part of the RING-finger domain E3 family (Petroski and Deshaies, 2005). Briefly, Cullin 1 acts as a scaffold on which SKP1 and RBX1 subunits assemble. SKP1 is involved in the recruitment of F box proteins (FBXW7, in the case of NICD), and RBX1 recruits a cognate E2 (Hao et al., 2007; Skaar et al., 2013). Fbxw7 consists of three isoforms (α, β, and γ) generated by alternative splicing and the isoform α, shown to ubiquitinate NICD, is localized to the nucleus (Matsumoto et al., 2006; O'neil et al., 2007; Welcker and Clurman, 2008; Crusio et al., 2010). Two domains are functionally important in the FBXW7 protein: the F-box domain, binding SKP1 (Bai et al., 1996), and the seven WD40 repeats mediating recognition/binding to the target protein in a specific consensus phospho-motif, the Cdc4 phospho-degron (Thr-Pro-Pro-Xaa-Ser, in which Thr and Ser residues are phosphorylated; Koepp et al., 2001; Welcker et al., 2003; Hao et al., 2007; Skaar et al., 2013; Figure 2D). A number of these phospho-degrons have been identified in the NICD PEST domain. Intriguingly, an additional hNICD1-specific degron has recently been identified within the N-terminal region, distinct from the PEST domain that is not recognized by FBXW7 (Broadus et al., 2016). Moreover, the E3 ligase, Itch, promoting PEST domain-independent NICD1 degradation (Qiu et al., 2000), does not mediate NICD1 degradation through the N1-Box (Broadus et al., 2016).

## NICD-FBXW7 interaction

Given the importance of Notch signaling in cell fate determination and cell cycle progression, it is not surprising that aberrations in the pathway lead to cancers and other diseases (Roy et al., 2007; Simpson et al., 2011; Wang et al., 2011; Kamath et al., 2012; Bolos et al., 2013; Huang et al., 2013; Lobry et al., 2013). Moreover, the pleiotropic nature of the pathway means the various Notch receptors can act as tumor suppressors for example in epithelial tumors or as oncogenes in leukemia and a variety of solid cancers (Radtke and Raj, 2003; Miele et al., 2006; Lobry et al., 2014; Alketbi and Attoub, 2015; Habets et al., 2015; Bonyadi Rad et al., 2016). From this vast literature we will focus here on activating mutations in Notch1 which are predominantly located in the extracellular heterodimerization (HD) domain resulting in ligand-independent exposure of the S2 cleavage site (Malecki et al., 2006; Van Tetering et al., 2009), or in the PEST domain, leading to constitutive activation of the pathway through increased NICD stability or in FBXW7, in line with its fundamental role in restricting the signaling strength/duration of the Notch pathway (Oberg et al., 2001; Tetzlaff et al., 2004; O'neil et al., 2007; Thompson et al., 2007; Wang et al., 2012; Bolos et al., 2013). For instance, *Notch1* mutations occur in over 50% of both pediatric and adult T-cell acute lymphoblastic leukemia (T-ALL) cases (Malyukova et al., 2007; Erbilgin et al., 2010), while *Fbxw7* mutations are found in up to 20% of T-ALL cases (Baldus et al., 2009; Mullighan, 2009). Furthermore, *Notch1* mutations were found in diffuse large B-cell lymphoma (DLBCL), splenic marginal zone lymphoma (SMZL), Hadju-Cheney syndrome (Isidor et al., 2011; Simpson et al., 2011; Kiel et al., 2012), breast cancer (Wang et al., 2015), and in 12% of non-small-cell lung carcinomas (NSCLCs), of which half were in the PEST domain (Westhoff et al., 2009). In these conditions, Notch target genes are highly upregulated.

Considering the variety of pathological conditions associated with alterations of NICD and FBXW7, there is a limited understanding of the regulation of this interaction. Our current understanding stems from a study on Sel-10, the nematode homolog of Fbxw7, showing the two proteins bind directly to each other and FBXW7 negatively regulates Notch signaling (Hubbard et al., 1997). Using cell models, human and murine homologs of Sel-10 were shown to play a key role in regulating Notch signaling by driving NICD to ubiquitin-proteasome mediated degradation (Gupta-Rossi et al., 2001; Oberg et al., 2001; Wu et al., 2001). NICD ubiquitination relies on the PEST domain. Studies on three NOTCH4 variants suggested that Sel-10 preferentially binds to phosphorylated forms of the C-terminal domain of NOTCH4 (Oberg et al., 2001; Wu et al., 2001). However, the NICD-Sel10 interaction has only been observed under overexpression conditions *in vitro*. It remains to be shown if this interaction occurs *in vivo*, if NICD interacts with any other E3 ligases, how this interaction is regulated and whether it is context-dependent. The *FBXW7* null mutant mice exhibit elevated levels of Notch4 intracellular domain and/or Notch1 intracellular domain alongside defects that are in alignment with a variety of roles identified for Notch in different developmental process such as cardiogenesis and vascular development. However, intriguingly, with respect to the segmentation clock, the absence of Fbxw7 seems to play a less major role in this process, at least according to the mutant phenotypes—although a detailed analysis has yet to be conducted (Tetzlaff et al., 2004; Tsunematsu et al., 2004). The results of these reports suggest that the mechanisms of NICD1 degradation during the somitogenesis process might actually rely on alternative (or redundant) mechanisms, highlighting again the need to further study alternative means of regulation of stability NICD1/degradation.

## Conclusions

In this review we provided a general overview of the critical role of Notch signaling in regulating the segmentation clock involved in somitogenesis. Notch activity is based on stability and turnover of its intracellular domain, NICD. This stability is regulated by phosphorylation of the PEST domain, targeting NICD to proteasome degradation upon recognition by the E3 ligase FBXW7. Mutations in the PEST domain, leading to aberrations in NICD stability, are the underlying cause of a number of solid and non-solid cancers and different genetic disorders. Therefore, uncovering the finer details of Notch pathway regulation merits attention, particularly because a wider comprehension of this process would provide further insights into the mechanisms involved in the onset of Notch-related diseases.

## Author contributions

FC and JD conceived the structure and content. FC wrote the initial draft document. FC designed and produced the figures. JD corrected and edited the document.

### Conflict of interest statement

The authors declare that the research was conducted in the absence of any commercial or financial relationships that could be construed as a potential conflict of interest.
